# Double-edged-sword effect of IL-1*β* on the osteogenesis of periodontal ligament stem cells via crosstalk between the NF-*κ*B, MAPK and BMP/Smad signaling pathways

**DOI:** 10.1038/cddis.2016.204

**Published:** 2016-07-14

**Authors:** C-y Mao, Y-g Wang, X Zhang, X-y Zheng, T-t Tang, E-y Lu

**Affiliations:** 1 Department of Prosthodontics, Shanghai Key Laboratory of Stomatology, Shanghai Ninth People's Hospital, Shanghai JiaoTong University School of Medicine, 639 Zhizaoju Road, Shanghai, China; 2 Shanghai Key Laboratory of Orthopaedic Implant, Department of Orthopaedics Surgery, Shanghai Ninth People's Hospital, Shanghai JiaoTong University School of Medicine, 639 Zhizaoju Road, Shanghai, China

## Abstract

Microenvironmental conditions can interfere with the functional role and differentiation of mesenchymal stem cells (MSCs). Recent studies suggest that an inflammatory microenvironment can significantly impact the osteogenic potential of periodontal ligament stem cells (PDLSCs), but the precise effects and mechanisms involved remain unclear. Here, we show for the first time that interleukin-1*β* (IL-1*β*) has dual roles in the osteogenesis of PDLSCs at concentrations ranging from physiologically healthy levels to those found in chronic periodontitis. Low doses of IL-1*β* activate the BMP/Smad signaling pathway to promote the osteogenesis of PDLSCs, but higher doses of IL-1*β* inhibit BMP/Smad signaling through the activation of nuclear factor-*κ*B (NF-*κ*B) and mitogen-activated protein kinase (MAPK) signaling, inhibiting osteogenesis. These results demonstrate that crosstalk between NF-*κ*B, MAPK and BMP/Smad signaling mediates this dual effect of IL-1*β* on PDLSCs. We also show that the impaired osteogenesis of PDLSCs results in more inflammatory cytokines and chemokines being released, inducing the chemotaxis of macrophages, which further clarifies the role of PDLSCs in the pathogenesis of periodontitis.

Approximately 90% of the population suffers from periodontitis,^1, 2^ which is characterized by chronic bacterial infections in the supporting structures of the teeth and a homeostatic imbalance between two coupled process in the periodontal system – bone resorption by osteoclasts and bone formation by osteoblasts. This disease involves interactions with bacterial products, numerous cell populations and different inflammatory mediators, and it can lead to tooth loss in adults.^1, 2^

Periodontal ligament stem cells (PDLSCs), a newly recognized sub-population of mesenchymal stem cells (MSCs), have attracted increasing attention in relation to their multipotency. As PDLSCs can easily be obtained from periodontal tissue, they are considered important for prospective cell-based therapies. Recently, PDLSCs have been shown to migrate to the site of periodontal lesions and to mediate periodontal regeneration.^3, 4, 5^ However, recent studies have found that the osteogenic capacity of stem cells is impaired in inflammatory microenvironments^6,7^ and that there are complex interactions between stem cells and the microenvironment under pathological conditions. Our previous studies found that disrupted and disease-associated microenvironments could influence the characteristics and functions of MSCs.^8-10^ Additionally, some studies have indicated that MSCs act in an immunomodulatory manner to regulate the function and chemotaxis of immune cells and that environmental factors may determine which immunomodulatory pathways are operational in MSCs.^11^ Thus, we assume that the mutual interactions between stem cells and inflammatory microenvironments are crucial to harnessing the regenerative potential of PDLSCs for therapeutic use.

Interleukin-1 (IL-1) is a pleiotropic cytokine and a central mediator of innate immunity and inflammation.^12^ In clinical studies, IL-1*β* has been found in increased concentrations in gingival crevicular fluid (GCF) and at sites of periodontal damage,^13, 14^ and levels of IL-1*β* have been reported to decrease after periodontal treatment.^15, 16^ Compared with levels at healthy sites, local IL-1*β* and tumor necrosis factor-*α* (TNF-*α*) levels in the microenvironments of chronic periodontitis have been found to be significantly elevated and to be associated with periodontal tissue destruction.^17–19^ IL-1 stimulates bone resorption by promoting osteoclast activation^17^^,^^20^^,21^ and mediates the osteoclastogenic effects of TNF-*α* by enhancing the expression of RANKL.^15^ In inflammatory microenvironments, IL-1 and TNF have a prominent role in the pathogenesis of periodontitis.^19^ Although TNF-*α* has activity similar to that of IL-1*β*, IL-1*β* is present at higher levels in inflamed gingival tissues, and its expression is limited to the connective tissue layer.^22^ Multiple studies have investigated the effect of IL-1*β* on osteoblast differentiation,^23, 24^ but conflicting data has been presented and the underlying mechanism of its effects remains unclear.^25^ A previous study has shown that the concentration of IL-1*β* in GCF is 145±167 pg/ml in healthy subjects and 6452±2289 pg/ml in patients with chronic periodontitis.^26^ In this study, we mimicked an inflammatory microenvironment using IL-1*β* at different concentrations that ranged from healthy physiological levels to those observed in the GCF in cases of chronic periodontitis^26^ and tried to establish an *in vitro* osteogenesis model to investigate the effects of different doses of IL-1*β* on PDLSCs.

Previously, it has been reported that the nuclear factor-*κ*B (NF-*κ*B) and mitogen-activated protein kinase (MAPK) signaling pathways have crucial roles in the regulation of inflammation and bone metabolism.^27^^28^ In addition, the BMP/Smad signaling pathways have important roles in the regulation of osteoblast differentiation.^29^ However, the roles these signaling pathways have in the osteogenesis of MSCs in inflammatory microenvironments remain unclear. In the present study, we investigated the interactions of BMP/Smad, MAPK and NF-*κ*B signaling pathways in mediating the IL-1*β*-regulated osteogenic differentiation of PDLSCs. Because the resident periodontal cells can produce various inflammatory mediators that induce inflammatory cells to invade the tissue and affect bone resorption,^30^ we further examined the role of PDLSCs in the pathogenesis of periodontitis by determining the production of inflammatory cytokines and chemokines by PDLSCs in which osteogenesis was inhibited by IL-1*β*.

## Results

### Identification of PDLSCs

We used flow cytometric analysis to characterize PDLSCs based on surface molecules. PDLSCs showed the characteristic pattern of mesenchymal surface markers, including STRO-1, CD146 and CD90, and negatively expressed the endothelial cell marker CD31 and the hematopoietic markers CD45 and CD14 (Supplementary Figure 1).

### Dual effect of IL-1*β* on the osteogenesis of PDLSCs

**Figure 1:**
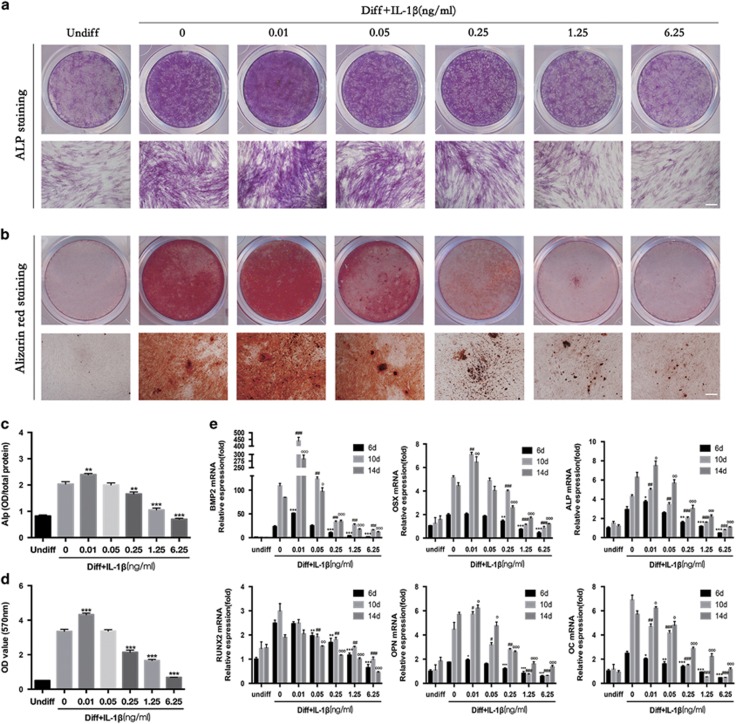
The dual effect of IL-1*β* on osteogenic differentiation of PDLSCs. Cells were incubated with osteogenic differentiation medium with or without the indicated concentrations of IL-1*β* (0.01–6.25 ng/ml). (**a**) Entire plate views and micrographs of alkaline phosphatase (ALP) staining at 7 days. (**b**) Entire plate views and micrographs of alizarin red staining at 21 days. Bars represent 100 *μ*m. (**c**) Quantitative evaluation of ALP activity. (**d**) Quantification of the alizarin red staining results. (**e**) BMP2, OSX, ALP, Runx2, OPN and OC mRNAs were subjected to real-time PCR analysis at 6, 10 and 14 days. The expression levels were normalized to that of *β*-actin. The data are presented as the mean±S.D. *P<0.05, **P<0.01, ***P<0.001 *versus* the Diff group (**c** and **d**) or the Diff group at 6 days (**e**), ^#^*P*<0.05, ^##^*P*<0.01, ^###^*P*<0.001 *versus* the Diff group at 10 days (**e**), ^○^*P*<0.05, ^○○^*P*<0.01, ^○○○^*P*<0.001 *versus* the Diff group at 14 days (**e**). All data were obtained from at least three independent experiments

The osteogenic differentiation of PDLSCs was induced by culturing them in osteogenic media with or without IL-1*β*. Both ALP staining (Figure 1a) at 7 days and alizarin red staining (Figure 1b) at 21 days indicated that IL-1*β* at 0.01 ng/ml stimulated the osteogenesis of PDLSCs, and IL-1*β* at 0.05 ng/ml showed no significant change of the osteogenesis of PDLSCs, whereas IL-1*β* at concentrations of 0.25, 1.25 and 6.25 ng/ml all clearly inhibited the osteogenic differentiation of PDLSCs. The quantitative data for ALP activity shown in Figure 1c and for alizarin red release shown in Figure 1d also indicate the different effects of IL-1*β* at different concentration on the osteogenesis of PDLSCs.

We then examined mRNA levels of *BMP2* and the osteoblast marker genes including *ALP, RUNX2, OSX, OPN* and *OC* at 6, 10 and 14 days. The transcription of *BMP2, OPN, ALP* (6, 10 and 14 days), *OC* (14 days) and *OSX* (10 and 14 days) increased by IL-1*β* at 0–0.01 ng/ml and was decreased by degrees by IL-1*β* at 0.05–6.25 ng/ml. *RUNX2* and *OC* (6 and 10 days) gradually decreased in response to IL-1*β* at 0–6.25 ng/ml in a dose-dependent manner (Figure 1e).

To assess whether changes in the expression levels of osteogenic markers might be a result of IL-1*β*-induced cell death, the viability of PDLSCs was determined via CCK8 analysis during the early stages of differentiation. CCK8 analysis showed no statistically significant changes during the early stages of differentiation (Supplementary Figure 2). Therefore, we conclude that the concentrations of IL-1*β* used in this study (0–6.25 ng/ml) had no cytotoxic effects on PDLSCs and that the effects on osteogenesis caused by IL-1*β* were not related to the physical–chemical precipitation that is usually observed during cell death in bone cell cultures.^31^

### Activation of NF-*κ*B and MAPK signaling in PDLSCs

**Figure 2:**
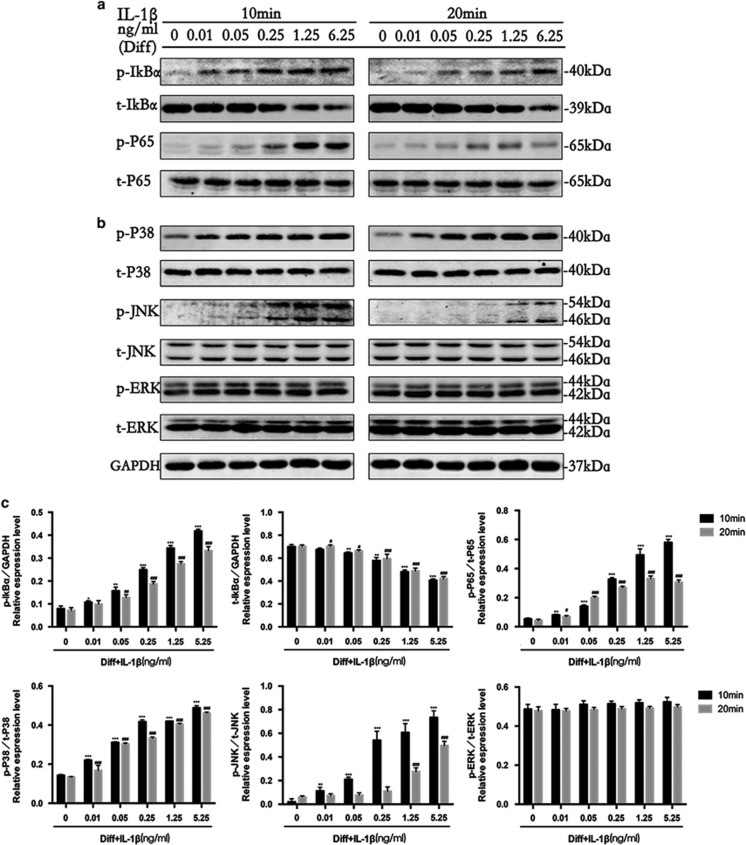
Expression characteristics of NF-*κ*B and MAPK signaling molecules during the osteogenic differentiation of PDLSCs with or without the indicated concentrations of IL-1β (0.01-6.25 ng/ml). (**a**) The levels of phosphorylated and overall IkBα and P65 at 10 min and 20 min were examined in whole cell lysates via western blotting. (**b**) The levels of phosphorylated and overall P38, JNK and ERK at 10 min and 20 min were examined in whole cell lysates via western blotting. (**c**) The average ratios of p-IkBα/GAPDH, t-IkBα/GAPDH, p-P65/P65, p-P38/P38, p-JNK/JNK and p-ERK/ERK were calculated based on the analysis the gray band intensities. The data are presented as the mean ± SD. *P<0.05, **P<0.01, ***P<0.001 versus the Diff group at 10 min. ^#^*P*<0.05, ^##^*P*<0.01, ^###^*P*<0.001 versus the Diff group at 20 min. All data were obtained from at least three independent experiments

To determine whether different doses of IL-1*β* affect PDLSCs differentiation through the NF-*κ*B and MAPK signaling pathways, we investigated the effects of the treatments using western blot analysis (Figures 2a and b) and quantified the resulting data (Figure 2c). IL-1*β* caused a rapid and strong activation of NF-*κ*B (Figure 2a) and MAPK (Figure 2b) signaling at 10 min and led to the dose-dependent phosphorylation of related proteins, including I*κ*B*α*, P65, P38 and JNK (c-Jun N-terminal kinase), but not included extracellular signal-regulated kinase (ERK). Furthermore, the phosphorylated level of proteins related to NF-*κ*B (I*κ*B*α* and P65) and MAPK (P38 and JNK) signally declined after 20 min. These data suggest that IL-1*β* may affect the osteogenesis of PDLSCs through NF-*κ*B and MAPK signaling.

### Activation of BMP/Smad signaling in PDLSCs

**Figure 3:**
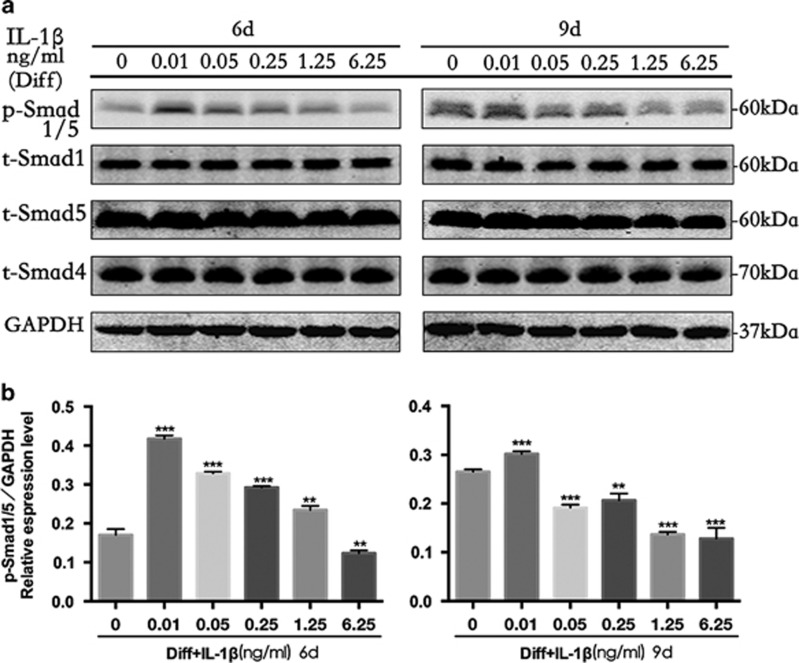
Expression characteristics of bone morphogenetic protein (BMP)/SMAD signaling molecules during the osteogenic differentiation of PDLSCs with or without the indicated concentrations of IL-1*β* (0.01–6.25 ng/ml). (**a**) The levels of phosphorylated Smad1/5 and overall Smad1/5 and Smad4 at 6 and 9 days were examined in whole-cell lysates via western blotting. (**b**) The average ratios of phosphorylated (p)-Smad1/5/GAPDH were calculated based on the analysis of the gray band intensities. The data are presented as the mean±SD. **P<0.01, ***P<0.001 *versus* the Diff group. All data were obtained from at least three independent experiments. GAPDH, glyceraldehyde-3-phosphate dehydrogenase

The effect of different doses of IL-1*β* on BMP/Smad signaling was determined via western blot analysis. As shown in Figure 1e, IL-1*β* activated BMP2 gene expression diversely, in which the transcription of BMP2 was significantly upregulated by IL-1*β* at 0–0.01 ng/ml and downregulated by IL-1*β* at 0.05–6.25 ng/ml. We also examined the activation of the downstream components of Smad signaling pathways during osteogenesis of PDLSCs. We found that a low dose of IL-1*β* (0.01 ng/ml) markedly promoted the phosphorylation of Smad1/5 at 6 days, whereas higher doses of IL-1*β* (0.05–6.25 ng/ml) showed a dose-dependent effect, resulting in a steady decrease in the phosphorylation level of Smad1/5 (Figure 3a). At 9 days, the phosphorylation level of Smad1/5 showed a slight increase in response to IL-1*β* at 0–0.01 ng/ml and gradually decreased with increasing IL-1*β* concentration (0.01–6.25 ng/ml) (Figure 3a). The quantified data are shown in Figure 3b. In addition, we found that the expression levels of Smad4 were not changed after stimulation by IL-1*β* (Figure 3a). Therefore, these findings suggest that IL-1*β* activates BMP/Smad at lower concentrations but inactivates these signal pathways at higher concentrations. This suggests crosstalk between BMP/Smad signaling and other mechanisms in the osteogenesis of PDLSCs.

### Inhibition of NF-*κ*B and MAPK rescues the impaired osteogenic differentiation caused by high-dose IL-1*β* inhibition

**Figure 4:**
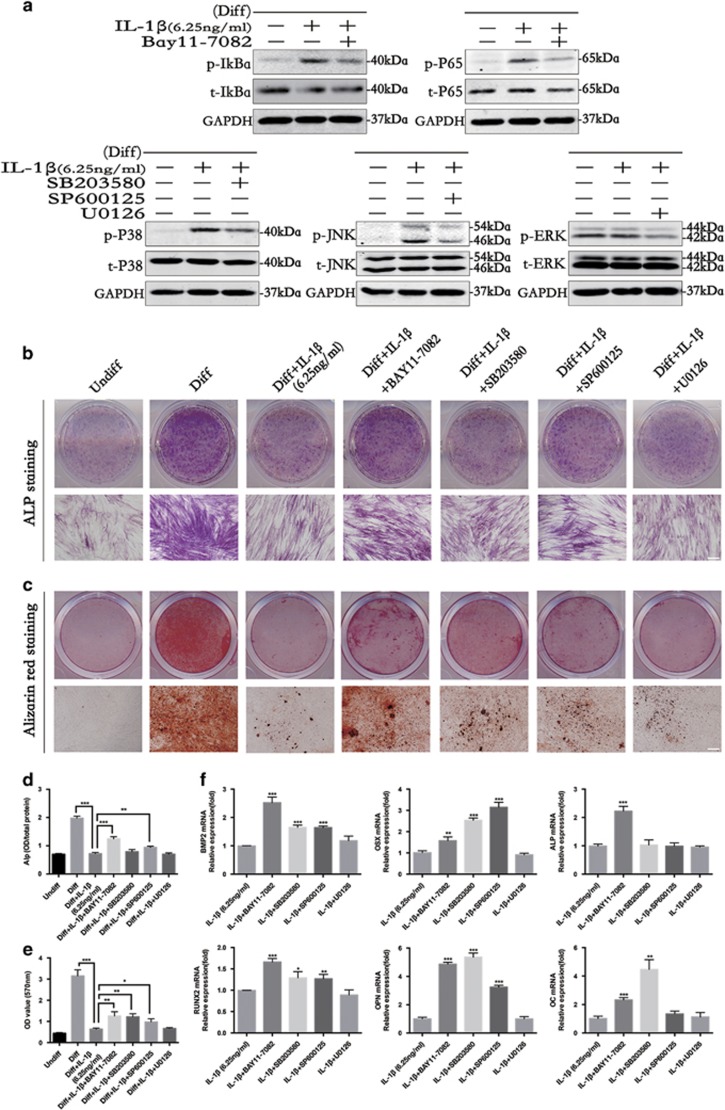
The effect of NF-*κ*B and MAPK inhibitors on the osteogenic differentiation of PDLSCs. Cells were incubated with osteogenic differentiation medium in the presence of IL-1*β* (6.25 ng/ml) along with an inhibitor of NF-*κ*B (Bay11-7085), p38 (SB203580), JNK (SP600125) or ERK (U0126). (**a**) Western blot analysis results for the levels of phosphorylated and overall I*κ*B*α*, P65, P38, JNK and ERK at 10 min. (**b**) Entire plate views and micrographs of alkaline phosphatase (ALP) staining at 7 days. (**c**) Entire plate views and micrographs of alizarin red staining at 21 days. Bars represent 100 *μ*m. (**d**) Quantitative evaluation of ALP activity. (**e**) Quantification of the alizarin red staining results. (**f**) BMP2, OSX, ALP, RUNX2, OPN and OC mRNAs was subjected to real-time PCR analysis at 10 days. The expression levels were normalized to that of *β*-actin. The data are presented as the mean±SD. *P<0.05, **P<0.01, ***P<0.001 versus the Diff+IL-1*β* (**d** and **e**) or IL-1*β* groups (**f**). All data were obtained from at least three independent experiments

To further clarify the role of NF-*κ*B and MAPK in osteoblast differentiation, we blocked the NF-*κ*B and MAPK signaling pathways using inhibitors of NF-*κ*B (BAY11-7082), P38 (SB203580), JNK (SP600125) and ERK (U0126). Western blot analysis was performed to determine the effective reduction of the phosphorylation levels of NF-*κ*B and MAPK after 10 min (Figure 4a). The quantified western blot results are shown in Supplementary Figure 3.

As shown in Figures 4b–e, blocking NF-*κ*B increased ALP and mineralization levels, and blocking MAPK increased the presence of mineralized nodules in PDLSCs. These results confirm that NF-*κ*B and MAPK have negative roles in the osteogenesis of PDLSCs. This observation was further supported by the real-time PCR results (Figure 4f), which show that blocking the activation of NF-*κ*B rescues the expression of BMP2 and osteoblast marker genes (*OSX, RUNX2, ALP, OC* and *OPN*) at 10 days, whereas blocking P38 signals rescues the production of *BMP2, OSX, RUNX2* and *OPN*. The inhibition of JNK upregulated the expression of *BMP2, OSX, RUNX2, OC* and *OPN*, whereas blocking the activation of ERK had no significant effect on the osteogenesis of PDLSCs under IL-1*β* stimulation.

### NF-*κ*B and P38/MAPK inhibition reactivates the BMP/Smad signaling downregulated by high-dose IL-1*β* treatment

**Figure 5:**
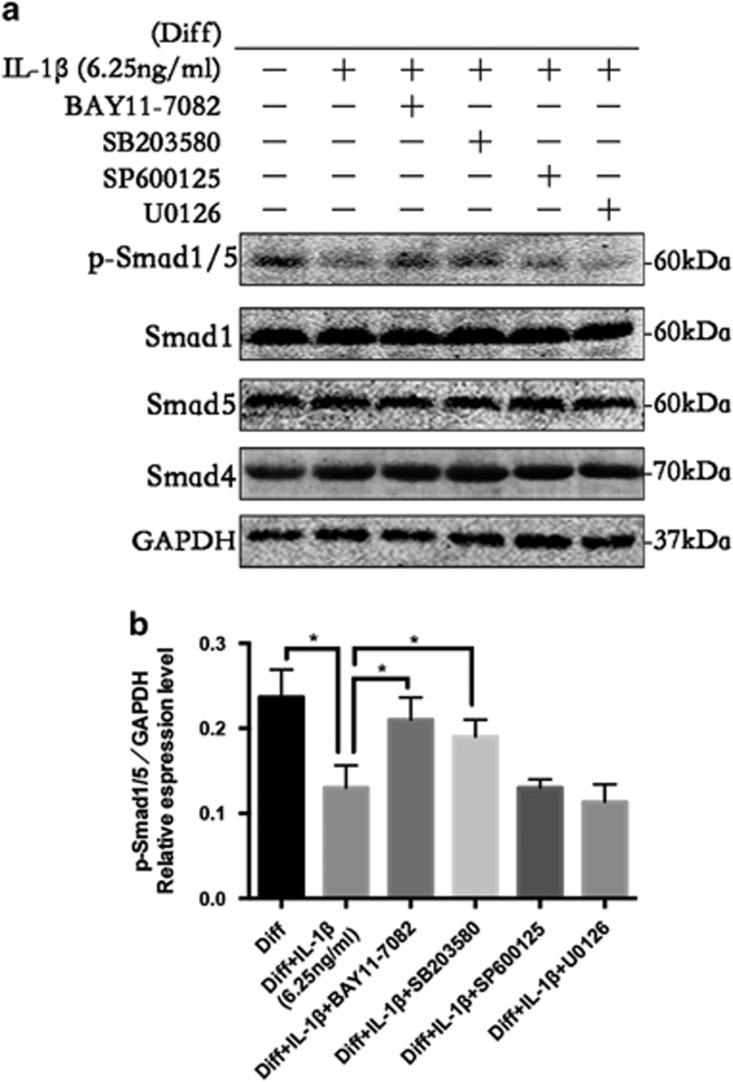
Expression characteristics of bone morphogenetic protein (BMP)/SMAD signaling molecules during the osteogenic differentiation of PDLSCs in the presence of IL-1*β* (6.25 ng/ml) and an inhibitor of NF-*κ*B (Bay11-7085), p38 (SB203580), JNK (SP600125) and ERK (U0126) at 9 days. (**a**) The levels of phosphorylated Smad1/5 and overall Smad1/5 and Smad4 at 9 days were examined in whole-cell lysates via western blotting. (**b**) The average ratios of phosphorylated (p)-Smad1/5/GAPDH were calculated based on the analysis of the gray band intensities. The data are presented as the mean±SD. *P<0.05 versus the Diff+IL-1*β* group. All data were obtained from at least three independent experiments

As reported previously, NF-*κ*B and MAPK signaling and BMP/Smad signaling have opposite biological functions during inflammatory and osteogenic processes.^27, 28, 32, 33^ Therefore, we hypothesized that NF-*κ*B and MAPK inhibit PDLSC osteogenesis through the inhibition of BMP/Smad signaling. To test this, specific NF-*κ*B and MAPK signaling inhibitors were used to inhibit the activation of NF-*κ*B and MAPK, and we observed the influence of this inhibition on BMP/Smad signaling activation via western blot analysis at 9 days. As shown in Figures 5a and b, the inhibition of NF-*κ*B and P38/MAPK led to a recovery of the Smad1/5 phosphorylation level, whereas blocking JNK and ERK showed no significant effect. Collectively, these results suggest a crosstalk between NF-*κ*B, MAPK and BMP/Smad signaling. NF-*κ*B and P38/MAPK signaling inhibited osteoblast differentiation of PDLSCs through the suppression of Smad1/5 phosphorylation, and NF-*κ*B and MAPK appear to act as pivotal mediators for the negative regulation of the osteogenesis of PDLSCs.

### BMP2 knockdown blocks osteogenic differentiation of PDLSCs induced by low-dose IL-1*β*

**Figure 6:**
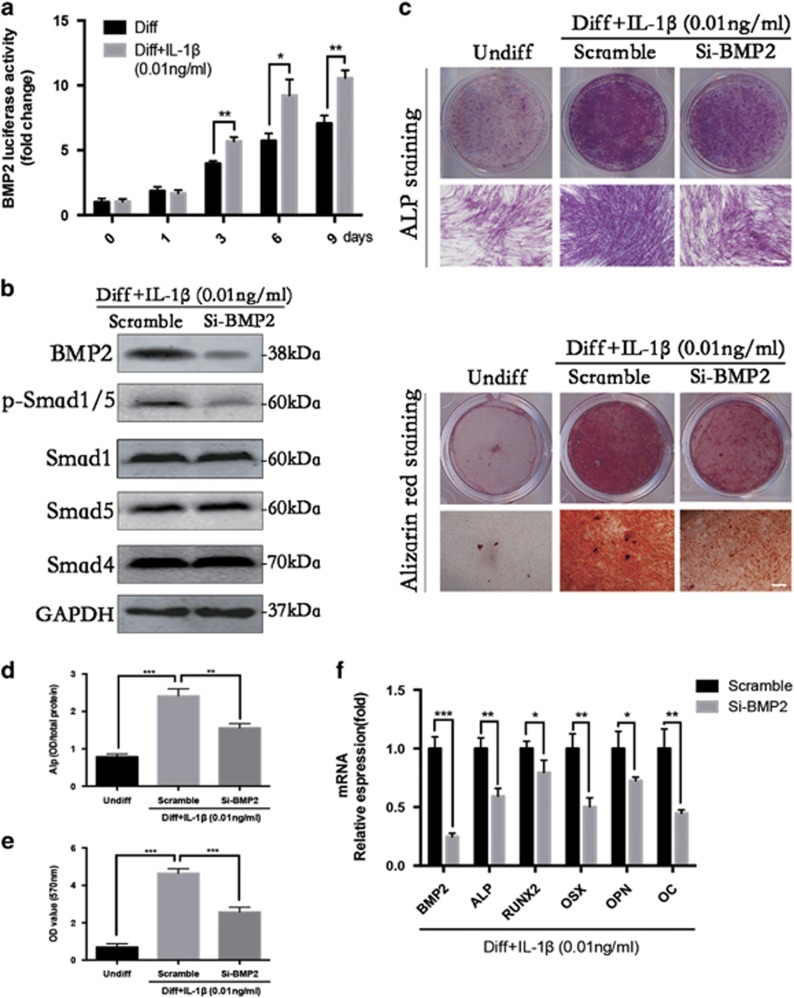
Bone morphogenetic protein 2 (BMP2) knockdown blocks osteogenic differentiation of PDLSCs induced by IL-1*β* (0.01 ng/ml). (**a**) The luciferase activities of BMP2 promoter were detected at 0, 1, 3, 6 and 9 days, and normalized to the negative control. (**b**) The levels of BMP2, phosphorylated Smad1/5 and overall Smad1/5 and Smad4 at 6 days were examined in whole-cell lysates via western blotting. (**c**) Entire plate views and micrographs of alkaline phosphatase (ALP) staining at 7 days and alizarin red staining at 21 days. Bars represent 100 *μ*m. (**d**) Quantitative evaluation of ALP activity. (**e**) Quantification of the alizarin red staining results. (**f**) BMP2, OSX, ALP, Runx2, OPN and OC mRNAs were subjected to real-time PCR analysis at 10 days. The expression levels were normalized to that of *β*-actin. The data are presented as the mean±S.D. *P<0.05, **P<0.01, ***P<0.001 versus the Diff (**a**) and Scramble group (**d**–**f**). All data were obtained from at least three independent experiments

We hypothesized that BMP2 has an important role in osteogenic differentiation of PDLSCs induced by low-dose IL-1*β*. To evaluate this hypothesis, we first analyzed the transcription of *BMP2* mRNA of PDLSCs treated by low-dose IL-1*β* by luciferase activity assay (Figure 6a). Compared with the control group, IL-1*β* (0.01 ng/ml) stimulated the transcription of *BMP* mRNA at 3, 6 and 9 days.

We then examined the effect of BMP2 using small interfering RNA (siRNA)-mediated gene silencing. As shown in Figure 6b, the western blot analysis show that BMP2 silencing markedly decreased the phosphorylation level of Smad1/5 at 6 days, and the quantified results are shown in Supplementary Figure 4. After BMP2 knockdown, ALP activity at 7 days (Figures 6c and d) and alizarin red staining at 21 days were inhibited (Figures 6c and e). The expression of osteoblast marker genes including *ALP, RUNX2, OSX, OPN* and *OC* were also inhibited at 10 days (Figure 6f). All these data suggested that BMP2 mediate the osteogenic differentiation of PDLSCs induced by low-dose IL-1*β*.

### IL-1*β* increases the chemotaxis of macrophages and cytokine production of PDLSCs

**Figure 7:**
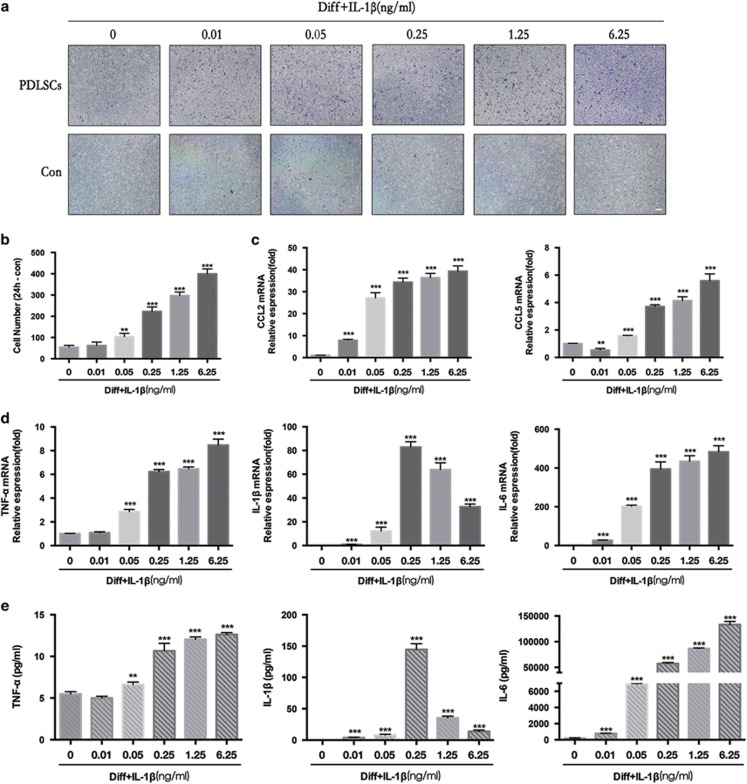
The immunomodulatory effects on osteogenic differentiation of PDLSCs with or without the indicated concentrations of IL-1*β* (0.01–6.25 ng/ml). (**a**) Transwells were used to determine the migration of RAW 264.7 macrophages at 24 h. Bars represent 100 *μ*m. (**b**) As in (**a**), the number of migrating cells were counted at × 200 magnification. (**c**) CCL2, CCL5 mRNAs of PDLSCs were subjected to real-time PCR analysis at 24 h. The expression levels were normalized to that of *β*-actin. (**d**) TNF-α, IL-1β and IL-6 mRNAs of PDLSCs were subjected to real-time PCR analysis at 24 h. The expression levels were normalized to that of *β*-actin. (**e**) Secreted concentrations of TNF-α, IL-1β and IL-6 in the culture supernatant of PDLSCs were measured via chemiluminescence cytokines analysis at 24 h. The secreted IL-1β was calculated as the supernatant concentration minus the initial concentration of IL-1β. The data are presented as the mean ± SD. *P<0.05, **P<0.01, ***P<0.001 versus the Diff group. All data were obtained from at least three independent experiments

To investigate the role of PDLSCs in inhibiting osteogenesis and their interplay with the inflammatory microenvironment in modulating the migration of macrophages, we performed a chemotaxis assay. First, we put PDLSCs (3 × 10^4^/well) in the lower transwell chamber. Osteogenic media with various concentrations of IL-1*β* (0–6.25 ng/ml) was added after incubated PDLSCs reached 80% confluence. Then, we seeded RAW 264.7 macrophages (3 × 10^5^/well) with culture media in the upper chamber of the transwell plates. After 24 h, we observed a dose-dependent increase in the number of cells that migrated from the top chamber to the bottom chamber. This demonstrates that when under inhibiting osteogenesis by IL-1*β*, PDLSCs promote the chemotaxis of RAW 264.7 macrophages (Figure 7a). The number of macrophages was counted and are shown in Figure 7b. To further determine how PDLSCs in which osteogenesis has been inhibited could affect the migration of macrophages and their role in inflammation, we next examined the chemokines and cytokines expressed at 24 h. The data show that the transcription of *CCL2* and *CCL5* markedly increased in a dose-dependent manner under IL-1*β* stimulation (0.05–6.25 ng/ml) (Figure 7c). The expression of inflammatory cytokines TNF-*α* and IL-6 increased in the same manner (Figures 7d and e), whereas the expression of IL-1*β* first markedly increased by the treatment of IL-1*β* at 0–0.25 ng/ml and then gradually decreased by IL-1*β* at 0.25–6.25 ng/ml, demonstrating that PDLSCs under inhibiting osteogenesis in inflammatory microenvironments increase macrophage infiltration and release more inflammatory mediators, which may contribute to bone resorption.

## Discussion

**Figure 8:**
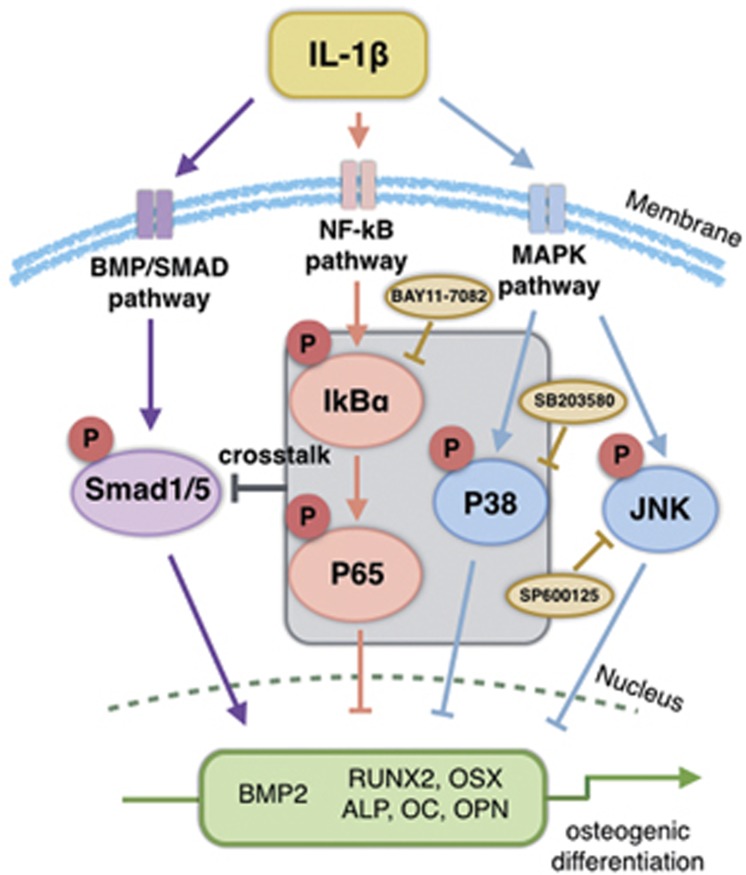
Schematic of the double-edged-sword effect of IL-1*β* on the osteogenesis of PDLSCs via crosstalk between NF-*κ*B, MAPK and bone morphogenetic protein (BMP)/Smad

This study investigated the role of the inflammatory environment and the possibility of crosstalk between the NF-*κ*B, MAPK and BMP/Smad signaling pathways in the regulation of osteogenic differentiation. We mimicked an inflammatory microenvironment by treating PDLSCs with IL-1*β* in concentrations ranging from healthy levels to levels found in chronic periodontitis and showed for the first time that (1) there is a dose-dependent dual role of IL-1*β* in modulating the osteogenesis of PDLSCs; (2) a low dose of IL-1*β* enhances osteoblast differentiation mainly through the BMP/Smad pathway, whereas higher doses of IL-1*β* exert inhibitory effects mainly through the NF-*κ*B and MAPK pathway; (3) crosstalk exits between the NF-*κ*B, MAPK and BMP/Smad signaling pathways that modulates the osteogenesis of PDLSCs; and (4) under conditions that inhibit osteogenesis, PDLSCs increase macrophage chemotaxis. (Figure 8).

It is commonly known that inflammatory cytokines act as negative modulators in the osteogenesis of resident cells. Some studies have reported that IL-1 inhibits the osteogenesis of MSCs or suppresses osteoblast-related gene expression.^7, 34^ However, other conflicting results indicate that IL-1*β* enhances the osteogenic potential of human MSCs.^24,^^35, 36^ It is possible that these opposite effects of IL-1*β* are related to differences in the source of MSCs and different IL-1*β* concentrations and exposure times. Surprisingly, we found a dual role of IL-1*β* in modulating the osteogenesis of PDLSCs. We demonstrated that IL-1*β* at lower concentrations (0–0.01 ng/ml) promotes the osteogenesis of PDLSCs, whereas IL-1*β* at higher concentrations (0.05–6.25 ng/ml) inhibits osteogenesis. We speculate that the inflammatory cytokines present in the microenvironment may serve as an important modulator of PDLSCs osteogenesis, and similar findings have also demonstrated that the IL-1*β* at the fracture site could modulate bone healing.^24^ Thus, targeting the inflammatory microenvironment could enhance the success of therapeutic approaches for the treatment of periodontitis using resident MSCs.

Downstream signals of the IL-1*β* pathway include the NF-*κ*B and MAPK signaling pathways. MAPK signals are regulated by a characteristic phosphorylation system in which a series of three protein kinases phosphorylate and activate one another. This signaling includes the ERK pathway, the JNK pathway and the P38 kinase pathway.^37^ The activation of NF-*κ*B has an important role in the onset of inflammation and cell proliferation or differentiation.^38^ We show here that the NF-*κ*B, P38/MAPK and JNK/MAPK pathways, but not the ERK/MAPK pathway, were gradually phosphorylated in a dose-dependent manner after a brief (10 min) treatment with IL-1*β*, whereas the inhibition of NF-*κ*B and MAPK using inhibitors of NF-*κ*B (BAY11-7082), P38 (SB203580) and JNK (SP600125) partly rescue the osteoblast differentiation that was impaired at higher levels of IL-1*β* (0.05–6.25 ng/ml).

BMP/Smad signaling pathways have important roles in the regulation of osteoblast differentiation,^29^ and this signaling can be influenced by inflammatory factors, including TNF-*α* and LPS.^27, 35^ Our findings show that low levels IL-1*β* (0.01 ng/ml) significantly increase the expression of BMP2 in PDLSCs and promote the activation of the BMP/Smad signaling pathway. This leads to the increased expression of osteoblast maker genes, including *OSX*, *ALP* and *OPN*, but not *RUNX2* and early *OC*. Some studies have indicated that BMP/Smad signaling can function independent of RUNX2, whereas the expression of OSX induced by BMP2 is mainly mediated by Dlx5, but not by RUNX2.^39^ However, compared with the effects of low levels of IL-1*β* (0.01 ng/ml), higher levels of IL-1*β* (0.05–6.25 ng/ml) inhibited BMP/Smad signaling in a dose-dependent manner.

It has been reported that BMP has biological functions that are opposite those of NF-*κ*B and MAPK during inflammatory processes.^27, 28, 32, 33^ We further investigated the potential crosstalk between BMP/Smad, NF-*κ*B and MAPK signaling. We suppressed NF-*κ*B and MAPK using specific inhibitors and found that the inhibition of NF-*κ*B and P38/MAPK partly restored the phosphorylation level of Smad1/5 under high-dose IL-1*β* treatment. Therefore, the dual roles IL-1*β* may be regulated by following mechanism: low-dose IL-1*β* treatment activates BMP/Smad signaling to promote the osteogenic differentiation of PDLSCs, whereas high-dose IL-1*β* treatment also activates the NF-*κ*B and MAPK pathway, which produce signals strong enough to suppress the phosphorylation of Smad1/5, leading to the inhibition of osteogenesis.

Studies of periodontal disease suggest a role for the chemokines CCL2 and CCL5 in macrophage migration,^40^ and cytokines such as TNF-*α*, IL-1*β* and IL-6 also contribute to osteoclast activation and inflammatory cell recruitment.^41, 42^ Our results show that coincident with impaired osteogenesis, IL-1*β*-treated PDLSCs could induce the chemotaxis of macrophages by expressing CCL2 and CCL5 and producing inflammatory cytokines, which helps to further explain the immunomodulatory effects of PDLSCs in the mediation of immune cells and the pathogenesis of periodontitis.

In summary, our study provides new insight into the double-edged-sword effect of IL-1*β* by showing that different concentrations of IL-1*β*, ranging from physiologically healthy levels to those observed in cases of chronic periodontitis, have different effects on the osteogenesis of PDLSCs via crosstalk between the NF-*κ*B, MAPK and BMP/Smad signaling pathways. Moreover, under conditions inhibiting osteogenesis, PDLSCs increase macrophage chemotaxis.

## Materials and Methods

### Samples, reagents and cell cultures

Human tooth samples were collected from five volunteer donors who received orthodontic treatment for a normal premolar tooth extraction in Shanghai Ninth People's Hospital. The patients were 18–22 years old and had no history of smoking or other contributory factors in their medical history. The experimental protocol was approved by the Ethics Committee of Shanghai JiaoTong University School of Medicine, and informed consent was obtained from all donors.

PDLSCs were isolated and cultured as described previously.^6, 7^ Briefly, the periodontal ligament (PDL) tissues were collected by scraping the root surface from the middle third to the apex, minced into 1 mm cubes and then placed into six-well culture dishes. The tissues were cultured in *α*-modified Eagle's minimum essential medium (*α*-MEM) (Hyclone, Logan, UT, USA) supplemented with 15% fetal bovine serum (Hyclone), 100 U/ml penicillin and 100 *μ*g/ml streptomycin at 37 °C. After a 2-week culture, cells from the PDL became subconfluent. To obtain homogenous populations of PDLSCs, single-cell-derived colony cultures were obtained using the limiting dilution technique as described previously,^43^ and these cultures were then mixed together. During cell passage, PDLSCs from different individuals were pooled. Multiple colony-derived PDLSCs at three to five passages were used, and PDLSCs were used at the same passage for each experiment.

RAW 264.7 cells were purchased from the American Type Culture Collection (Rockville, MD, USA). *α*-MEM and fetal bovine serum (FBS) were purchased from Hyclone. IL-1*β* was purchased from PeproTech (Rocky Hill, NJ, USA). P38 (SB203580; Sigma-Aldrich, St. Louis, MO, USA), JNK (SP600125; Sigma-Aldrich) and ERK (U0126; Cell Signaling Technology, Boston, MA, USA) inhibitors (all at 10 *μ*M) and an NF-*κ*B inhibitor (BAY11-7082; Sigma-Aldrich) (5 *μ*M) were used in this study.

### Flow cytometry

To identify MSC phenotypes, ~1 × 10^6^ PDLSCs were washed in phosphate-buffered saline (PBS) and then incubated with the following mouse anti-human monoclonal antibodies: CD146 (FITC), CD31 (Alexa Fluor 488), CD90 (PE), CD14(PE), CD45 (APC) (eBioscience, San Diego, CA, USA) and STRO-1(Alexa Fluor 647) (BioLegend, San Diego, CA, USA). Cells were incubated for 45 min at room temperature. The cell suspensions were then washed three times with PBS and analyzed using a flow cytometer (FACSCalibur; BD Biosciences, Franklin Lakes, NJ, USA).

### Cell viability assay

The cytotoxic effects of IL-1*β* were determined using a CCK8 assay according to the manufacturer's instructions. PDLSCs were plated in 96-well plates at a density of 4 × 10^3^ cells per well and cultured in *α*-MEM with 10% FBS for 24 or 48 h. Cells were then treated with different concentrations of IL-1*β* (0, 0.01, 0.05, 0.25, 0.125 or 0.625 ng/ml) for 24 h. Ten microliters of CCK8 buffer were added to each well, and cells were incubated at 37 °C for an additional 2 h. The absorbance was then measured at a wavelength of 450 nm (650 nm reference) on an ELX800 absorbance microplate reader (Bio-Tek, Winooski, VT, USA). Cell viability was calculated relative to a control using the following formula: (experimental group OD−zeroing OD)/(control group OD−zeroing OD).

### Construction of luciferase reporter gene and luciferase assay

PDLSCs were transfected using Lipofectamine 2000 (Invitrogen, Carlsbad, CA, USA) following the manufacturer's instructions. The BMP2 promoter-luciferase reporter construct, consisting of 1.1 kb of BMP2 5′-flanking sequence cloned in front of the Gaussia luciferase gene, was purchased from Genecopoeia (Rockville, MD, USA). As a negative control, a subset of cells was transfected with the reporter vector without promoter sequence. Secreted Gaussia luciferase was assayed from culture media using the Secrete-Pair Luminescence Assay Kit (Genecopoeia), according to the manufacturer's instructions. Luciferase activity was normalized to the negative control.

### siRNA transfection

BMP2-specific and -nonspecific siRNA duplexes with Chol-OMe-Cy5 modified were synthesized by Ribobio Co. Ltd (Guangzhou, China). The sequences of siRNA oligonucleotides are described as follows: si-BMP2, 5′-GAAACGAGUGGGAAAACAA dTdT-3′ scrambled nontargeting siRNA, 5′-CGUACGCGGAAUACUUCGA dTdT-3′. The transfection of cells was performed according to the manufacturer's protocol (Ribobio, Guangzhou, China).

### Osteogenic differentiation

A total of 3 × 10^4^ PDLSCs were plated into each well of 24-well plates and cultured. To induce osteoblast differentiation, after reaching 80% confluence, PDLSCs were cultured in *α*-MEM supplemented with 10% FBS, 50 *μ*g/ml of ascorbic acid and 5 mM *β*-glycerophosphate for 7–21 days. The medium was changed every 2 days. Cells were washed two times in PBS after fixation in 4% paraformaldehyde for 20 min. Calcium accumulation was detected via 2% alizarin red staining (pH 4.2), and calcium levels were then measured using 100 mM cetylpyridinium chloride for 10 min to solubilize and release the calcium-bound alizarin red into solution. The data are expressed as the absorbance at 570 nm of the released alizarin red (detected using a ELX800 absorbance microplate reader).^44^ ALP staining and ALP activity were determined using an Alkaline Phosphatase Color Development Kit (Hongqiao, Shanghai, China) and an Alkaline Phosphatase Detection Kit (Jiancheng Bioengineering, Nanjing, China; http://www.njjcbio.com) according to the manufacturer's suggested protocol.

### Real-time PCR

Total RNA was extracted using the Qiagen RNeasy Mini Kit (Qiagen, Valencia, CA, USA) according to the manufacturer's instructions, and cDNA was synthesized from 1 *μ*g of total RNA using reverse transcriptase (TaKaRa Biotechnology, Otsu, Japan). Real-time PCR was performed using the SYBR Premix Ex Taq Kit (TaKaRa) with the ABI Prism 7500 Fast Real-Time PCR System (Applied Biosystems, Foster City, CA, USA) according to the manufacturer's instructions. The primers were designed and selected using BLAST. Gene expression was measured using the DDCt method. *β*-Actin was used as the internal control. The primer sequences are summarized in Supplementary Table 1.

### Western blot analysis

The cells were washed two times with cold PBS, and the total protein was extracted using an RIPA lysis buffer. The protein concentration was quantified using a BCA Protein Assay Kit (Thermo Scientific, Rockford, IL, USA), and proteins were separated via 10% SDS-PAGE and transferred onto polyvinylidene fluoride membranes (Millipore, Billerica, MA, USA). The transfer membranes were blocked with 5% fat-free milk at room temperature for 1 h and then incubated with primary antibodies against BMP2 (1 : 1000; Abcam, Cambridge, UK), ERK1/2, p-ERK1/2, JNK, p-JNK, p38, p-p38, I*κ*B*α*, p-I*κ*B*α*, p65, p-p65, p-Smad1/5, Smad1/5 and Smad4 (1 : 1000; Cell Signaling Technology) at 4 °C overnight. After three washes, the membranes were incubated with appropriate secondary antibodies that were conjugated with IRDye 800CW at room temperature for 1 h. Immunoreactive bands were detected using the Odyssey infrared imaging system (LI-COR, Lincoln, NE, USA). The GAPDH antibody (1 : 1000; Cell Signaling Technology) was used as a control. The intensity of each band was analyzed using the ImageJ software (Bethesda, MD, USA).

### Transwell assay

PDLSCs were plated at 3 × 10^4^ cells per well in the lower transwell chamber and cultured to 80% confluence in culture medium (*α*-MEM with 10% FBS and 1% antibiotic). The medium was replaced with an osteogenic medium containing 0, 0.01, 0.05, 0.25, 1.25 or 6.25 ng/ml IL-1*β* and cells were cultured for 24 h. An osteogenic medium with 0, 0.01, 0.05, 0.25, 1.25 or 6.25 ng/ml IL-1*β* and without PDLSCs used as a control. A total of 3 × 10^4^ RAW 264.7 cells were plated on a Matrigel-coated polycarbonate membrane insert (8.0 mm pores) in a transwell apparatus (Costar, Shanghai, China) and maintained in 100 μl of complete medium (*α*-MEM with 10% FBS and 1% antibiotic). Then, the inserts were washed with PBS, and the cells on the top surface of the insert were removed by wiping the surfaces with a cotton swab. The cells that migrated to the bottom surface of the insert were fixed with 4% paraformaldehyde, stained with 0.1% crystal violet and then subjected to a microscopic inspection and cell count. The cells were counted under × 200 magnification. Every sample was counted in five randomly chosen fields and the values were averaged.

### Cytokine analysis

PDLSCs were seeded at 3 × 10^4^ cells per well in 24-well plates with culture medium (*α*-MEM with 10% FBS and 1% antibiotic). After 80% confluence was reached, the medium was replaced with an osteogenic medium containing 0, 0.01, 0.05, 0.25, 1.25 or 6.25 ng/ml IL-1*β*, and the cells were cultured for 24 h. The production of TNF-*α*, IL-1*β* and IL-6 in the supernatant was measured with TNF-*α*, IL-1*β* and IL-6 Chemiluminescence Analysis Kits (Siemens Healthcare Diagnostics Inc.) using an IMMULITE/IMMULITE 1000 System (Siemens Healthcare Diagnostics Inc., Marburg, Germany), following the manufacturer's instructions. In all procedures, contamination and exposure to direct sunlight were avoided, as per the manufacturer's guidelines.

### Statistical analysis

The data are expressed as the mean±S.D. from at least three independent experiments. The results were analyzed via Student's *t*-test or one-way analysis of variance) using the SPSS 13.0 software (SPSS Inc., Chicago, IL, USA). *P*<0.05 indicated a significant difference between groups.

## Abbreviations

MSC: mesenchymal stem cell
PDLSC: periodontal ligament stem cell
IL-1*β*: interleukin-1*β*
NF-*κ*B: nuclear factor-*κ*B
MAPK: mitogen-activated protein kinase
GCF: gingival crevicular fluid
TNF-*α*: tumor necrosis factor-*α*

## References

[bib1] Burt B. Position paper: epidemiology of periodontal diseases. J Periodontol 2005; 76: 1406–1419. 16101377 10.1902/jop.2005.76.8.1406

[bib2] Papapanou PN. The prevalence of periodontitis in the US: forget what you were told. J Dent Res 2012; 91: 907–908. 22935674 10.1177/0022034512458692

[bib3] Liu Y, Zheng Y, Ding G, Fang D, Zhang C, Bartold PM et al. Periodontal ligament stem cell-mediated treatment for periodontitis in miniature swine. Stem Cells 2008; 26: 1065–1073. 18238856 10.1634/stemcells.2007-0734 PMC2653213

[bib4] Kunze M, Huber A, Krajewski A, Lowden E, Schuhmann N, Buening H et al. Efficient gene transfer to periodontal ligament cells and human gingival fibroblasts by adeno-associated virus vectors. J Dent 2009; 37: 502–508. 19362764 10.1016/j.jdent.2009.03.001

[bib5] Huang GT, Gronthos S, Shi S. Mesenchymal stem cells derived from dental tissues vs. those from other sources: their biology and role in regenerative medicine. J Dent Res 2009; 88: 792–806. 19767575 10.1177/0022034509340867 PMC2830488

[bib6] Park JC, Kim JM, Jung IH, Kim JC, Choi SH, Cho KS et al. Isolation and characterization of human periodontal ligament (PDL) stem cells (PDLSCs) from the inflamed PDL tissue: *in vitro* and *in vivo* evaluations. J Clin Periodontol 2011; 38: 721–731. 21449989 10.1111/j.1600-051X.2011.01716.x

[bib7] Kong X, Liu Y, Ye R, Zhu B, Zhu Y, Liu X et al. GSK3beta is a checkpoint for TNF-alpha-mediated impaired osteogenic differentiation of mesenchymal stem cells in inflammatory microenvironments. Biochim Biophys Acta 2013; 1830: 5119–5129. 23911749 10.1016/j.bbagen.2013.07.027

[bib8] Bian ZY, Fan QM, Li G, Xu WT, Tang TT. Human mesenchymal stem cells promote growth of osteosarcoma: involvement of interleukin-6 in the interaction between human mesenchymal stem cells and Saos-2. Cancer Sci 2010; 101: 2554–2560. 20874851 10.1111/j.1349-7006.2010.01731.x PMC11159660

[bib9] Fan QM, Yue B, Bian ZY, Xu WT, Tu B, Dai KR et al. The CREB-Smad6-Runx2 axis contributes to the impaired osteogenesis potential of bone marrow stromal cells in fibrous dysplasia of bone. J Pathol 2012; 228: 45–55. 22450860 10.1002/path.4033

[bib10] Tu B, Peng ZX, Fan QM, Du L, Yan W, Tang TT. Osteosarcoma cells promote the production of pro-tumor cytokines in mesenchymal stem cells by inhibiting their osteogenic differentiation through the TGF-beta/Smad2/3 pathway. Exp Cell Res 2014; 320: 164–173. 24183998 10.1016/j.yexcr.2013.10.013

[bib11] Hoogduijn MJ, Popp F, Verbeek R, Masoodi M, Nicolaou A, Baan C et al. The immunomodulatory properties of mesenchymal stem cells and their use for immunotherapy. Int Immunopharmacol 2010; 10: 1496–1500. 20619384 10.1016/j.intimp.2010.06.019

[bib12] Garlanda C, Dinarello CA, Mantovani A. The interleukin-1 family: back to the future. Immunity 2013; 39: 1003–1018. 24332029 10.1016/j.immuni.2013.11.010 PMC3933951

[bib13] Thunell DH, Tymkiw KD, Johnson GK, Joly S, Burnell KK, Cavanaugh JE et al. A multiplex immunoassay demonstrates reductions in gingival crevicular fluid cytokines following initial periodontal therapy. J Periodont Res 2010; 45: 148–152. 19602112 10.1111/j.1600-0765.2009.01204.x PMC5405696

[bib14] Cetinkaya B, Guzeldemir E, Ogus E, Bulut S. Proinflammatory and anti-inflammatory cytokines in gingival crevicular fluid and serum of patients with rheumatoid arthritis and patients with chronic periodontitis. J Periodontol 2013; 84: 84–93. 22414257 10.1902/jop.2012.110467

[bib15] Wei S, Kitaura H, Zhou P, Ross FP, Teitelbaum SL. IL-1 mediates TNF-induced osteoclastogenesis. J Clin Invest 2005; 115: 282–290. 15668736 10.1172/JCI23394 PMC544608

[bib16] Holmlund A, Hanstrom L, Lerner UH. Bone resorbing activity and cytokine levels in gingival crevicular fluid before and after treatment of periodontal disease. J Clin Periodontol 2004; 31: 475–482. 15142219 10.1111/j.1600-051X.2004.00504.x

[bib17] Schett G. Effects of inflammatory and anti-inflammatory cytokines on the bone. Eur J Clin Invest 2011; 41: 1361–1366. 21615394 10.1111/j.1365-2362.2011.02545.x

[bib18] Ertugrul AS, Sahin H, Dikilitas A, Alpaslan N, Bozoglan A. Comparison of CCL28, interleukin-8, interleukin-1beta and tumor necrosis factor-alpha in subjects with gingivitis, chronic periodontitis and generalized aggressive periodontitis. Journal of periodontal research 2013; 48: 44–51. 22812409 10.1111/j.1600-0765.2012.01500.x

[bib19] Graves DT, Cochran D. The contribution of interleukin-1 and tumor necrosis factor to periodontal tissue destruction. Journal of periodontology 2003; 74: 391–401. 12710761 10.1902/jop.2003.74.3.391

[bib20] Pfeilschifter J, Chenu C, Bird A, Mundy GR, Roodman GD. Interleukin-1 and tumor necrosis factor stimulate the formation of human osteoclastlike cells *in vitro*. J Bone Miner Res 1989; 4: 113–118. 2785743 10.1002/jbmr.5650040116

[bib21] Braun T, Zwerina J. Positive regulators of osteoclastogenesis and bone resorption in rheumatoid arthritis. Arthritis Res Ther 2011; 13: 235. 21861862 10.1186/ar3380 PMC3239343

[bib22] Stashenko P, Jandinski JJ, Fujiyoshi P, Rynar J, Socransky SS. Tissue levels of bone resorptive cytokines in periodontal disease. J Periodontol 1991; 62: 504–509. 1920018 10.1902/jop.1991.62.8.504

[bib23] Sonomoto K, Yamaoka K, Oshita K, Fukuyo S, Zhang X, Nakano K et al. Interleukin-1beta induces differentiation of human mesenchymal stem cells into osteoblasts via the Wnt-5a/receptor tyrosine kinase-like orphan receptor 2 pathway. Arthritis Rheum 2012; 64: 3355–3363. 22674197 10.1002/art.34555

[bib24] Mumme M, Scotti C, Papadimitropoulos A, Todorov A, Hoffmann W, Bocelli-Tyndall C et al. Interleukin-1beta modulates endochondral ossification by human adult bone marrow stromal cells. Eur Cell Mater 2012; 24: 224–236. 23007908 10.22203/ecm.v024a16

[bib25] Deshpande S, James AW, Blough J, Donneys A, Wang SC, Cederna PS et al. Reconciling the effects of inflammatory cytokines on mesenchymal cell osteogenic differentiation. J Surg Res 2013; 185: 278–285. 23972621 10.1016/j.jss.2013.06.063 PMC4489542

[bib26] Luo L, Xie P, Gong P, Tang XH, Ding Y, Deng LX. Expression of HMGB1 and HMGN2 in gingival tissues, GCF and PICF of periodontitis patients and peri-implantitis. Archiv Oral Biol 2011; 56: 1106–1111. 10.1016/j.archoralbio.2011.03.020 21570059

[bib27] Huang RL, Yuan Y, Zou GM, Liu G, Tu J, Li Q. LPS-stimulated inflammatory environment inhibits BMP-2-induced osteoblastic differentiation through crosstalk between TLR4/MyD88/NF-kappaB and BMP/Smad signaling. Stem Cells Dev 2014; 23: 277–289. 24050190 10.1089/scd.2013.0345 PMC3904516

[bib28] Daigang L, Jining Q, Jinlai L, Pengfei W, Chuan S, Liangku H et al. LPS-stimulated inflammation inhibits BMP-9-induced osteoblastic differentiation through crosstalk between BMP/MAPK and Smad signaling. Exp Cell Res 2016; 341: 54–60. 26794904 10.1016/j.yexcr.2016.01.009

[bib29] Chen G, Deng C, Li YP. TGF-beta and BMP signaling in osteoblast differentiation and bone formation. Int J Biol Sci 2012; 8: 272–288. 22298955 10.7150/ijbs.2929 PMC3269610

[bib30] Yucel-Lindberg T, Bage T. Inflammatory mediators in the pathogenesis of periodontitis. Expert Rev Mol Med 2013; 15: e7. 23915822 10.1017/erm.2013.8

[bib31] Magne D, Bluteau G, Faucheux C, Palmer G, Vignes-Colombeix C, Pilet P et al. Phosphate is a specific signal for ATDC5 chondrocyte maturation and apoptosis-associated mineralization: possible implication of apoptosis in the regulation of endochondral ossification. J Bone Miner Res 2003; 18: 1430–1442. 12929932 10.1359/jbmr.2003.18.8.1430 PMC2071932

[bib32] Hirata-Tsuchiya S, Fukushima H, Katagiri T, Ohte S, Shin M, Nagano K et al. Inhibition of BMP2-induced bone formation by the p65 subunit of NF-kappaB via an interaction with Smad4. Mol Endocrinol 2014; 28: 1460–1470. 25029242 10.1210/me.2014-1094 PMC5414795

[bib33] Yamazaki M, Fukushima H, Shin M, Katagiri T, Doi T, Takahashi T et al. Tumor necrosis factor alpha represses bone morphogenetic protein (BMP) signaling by interfering with the DNA binding of Smads through the activation of NF-kappaB. J Biol Chem 2009; 284: 35987–35995. 19854828 10.1074/jbc.M109.070540 PMC2791026

[bib34] Polzer K, Joosten L, Gasser J, Distler JH, Ruiz G, Baum W et al. Interleukin-1 is essential for systemic inflammatory bone loss. Ann Rheum Dis 2010; 69: 284–290. 19196726 10.1136/ard.2008.104786

[bib35] Loebel C, Czekanska EM, Staudacher J, Salzmann G, Richards RG, Alini M et al. The calcification potential of human MSCs can be enhanced by interleukin-1beta in osteogenic medium. J Tissue Eng Regen Med 2014 (doi:10.1002/term.1950). 10.1002/term.1950 25185894

[bib36] Ding J, Ghali O, Lencel P, Broux O, Chauveau C, Devedjian JC et al. TNF-alpha and IL-1beta inhibit RUNX2 and collagen expression but increase alkaline phosphatase activity and mineralization in human mesenchymal stem cells. Life Sci 2009; 84: 499–504. 19302812 10.1016/j.lfs.2009.01.013

[bib37] Johnson GL, Lapadat R. Mitogen-activated protein kinase pathways mediated by ERK, JNK, and p38 protein kinases. Science 2002; 298: 1911–1912. 12471242 10.1126/science.1072682

[bib38] Viatour P, Merville MP, Bours V, Chariot A. Phosphorylation of NF-kappaB and IkappaB proteins: implications in cancer and inflammation. Trends Biochem Sci 2005; 30: 43–52. 15653325 10.1016/j.tibs.2004.11.009

[bib39] Lee MH, Kwon TG, Park HS, Wozney JM, Ryoo HM. BMP-2-induced Osterix expression is mediated by Dlx5 but is independent of Runx2. Biochem Biophys Res Commun 2003; 309: 689–694. 12963046 10.1016/j.bbrc.2003.08.058

[bib40] Silva TA, Garlet GP, Fukada SY, Silva JS, Cunha FQ. Chemokines in oral inflammatory diseases: apical periodontitis and periodontal disease. J Dent Res 2007; 86: 306–319. 17384024 10.1177/154405910708600403

[bib41] Bartold PM, Cantley MD, Haynes DR. Mechanisms and control of pathologic bone loss in periodontitis. Periodontology 2000 2010; 53: 55–69. 20403105 10.1111/j.1600-0757.2010.00347.x

[bib42] Deo V, Bhongade ML. Pathogenesis of periodontitis: role of cytokines in host response. Dentistry today 2010; 29: 60–62, 4–6; quiz 8–9. 20973418

[bib43] Huo N, Tang L, Yang Z, Qian H, Wang Y, Han C et al. Differentiation of dermal multipotent cells into odontogenic lineage induced by embryonic and neonatal tooth germ cell-conditioned medium. Stem Cells Dev 2010; 19: 93–104. 19469666 10.1089/scd.2009.0048

[bib44] Johnson K, Hashimoto S, Lotz M, Pritzker K, Goding J, Terkeltaub R. Up-regulated expression of the phosphodiesterase nucleotide pyrophosphatase family member PC-1 is a marker and pathogenic factor for knee meniscal cartilage matrix calcification. Arthritis Rheum 2001; 44: 1071–1081. 11352238 10.1002/1529-0131(200105)44:5<1071::AID-ANR187>3.0.CO;2-3

