# Supplementary material for: Double-edged-sword effect of IL-1β on the osteogenesis of periodontal ligament stem cells via crosstalk between the NF-κB, MAPK and BMP/Smad signaling pathways
Source: Cell Death Dis. 2016 Jul 14;7(7):e2296–. doi: 10.1038/cddis.2016.204 (PMC4973347; doi:10.1038/cddis.2016.204)
Supplement: Supplementary Information [file cddis2016204x1.pdf]

Supplementary Fig. 1.

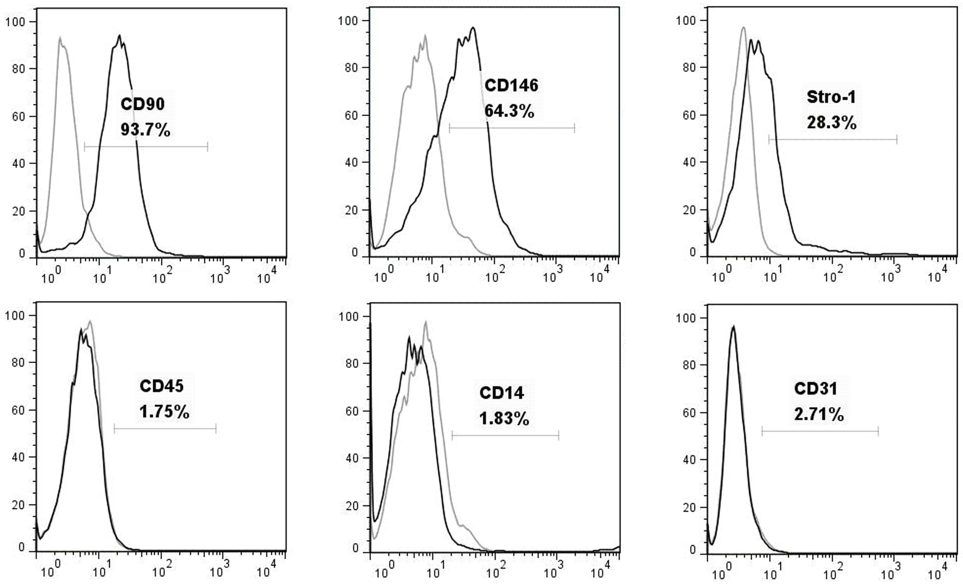

Flow cytometric characterization of surface marker expression patterns on PDLSCs. Cells were positive for CD90, CD146 and STRO-1 but negative for CD45, CD14 (hematopoietic stem cells) and CD31 (endothelial cells).

Supplementary Fig. 2.

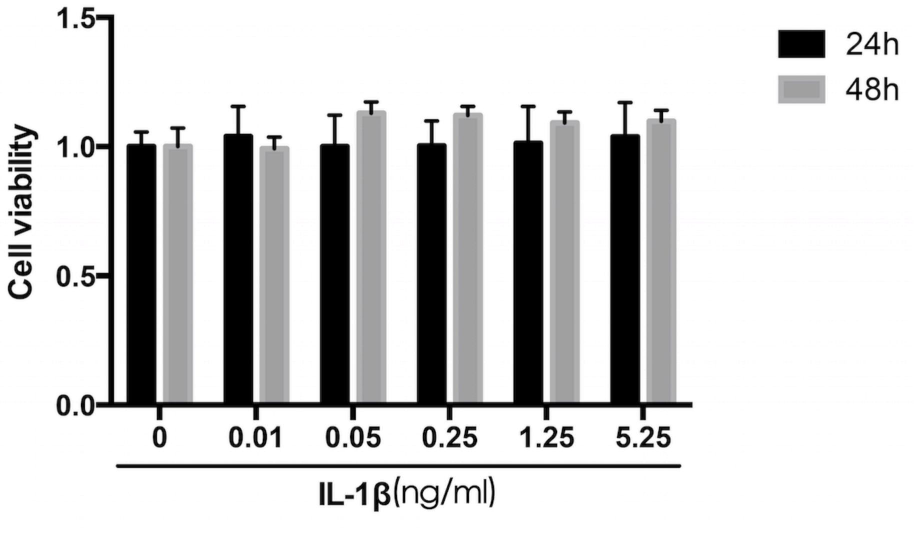

IL-1β treatment did not affect the viability of PDLSCs. The cell viability assay was performed at 24 h and 48 h in the presence of different concentrations of IL-1β. The data are presented as the mean ± SD. All data were obtained from at least three independent experiments.

Supplementary Fig. 3.

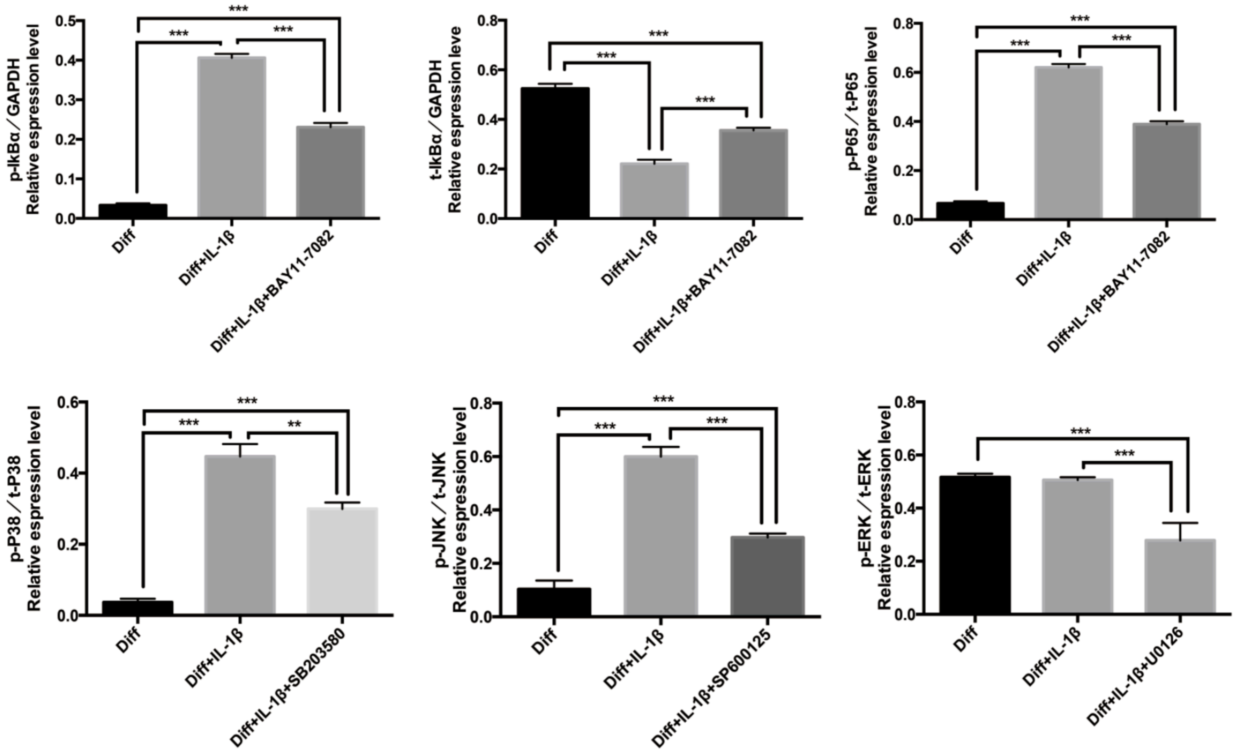

The quantification of NF-κB and MAPK inhibitors on the activation of NF-κB and MAPK signaling. Cells were incubated with osteogenic differentiation medium in the presence of IL-1β (6.25 ng/ml) along with the inhibitor of NF-κB (Bay11–7085), p38 (SB203580), JNK (SP600125), or ERK (U0126). The average ratios of p-IkBα/GAPDH, t-IkBα/GAPDH, p-P65/P65, p-P38/P38, p-JNK/JNK and p-ERK/ERK were calculated based on the analysis of the gray band intensities in western blotting at 10 min. The data are presented as the mean ± SD. **P<0.01, ***P<0.001 versus each group. All data were obtained from at least three independent experiments.

Supplementary Fig. 4.

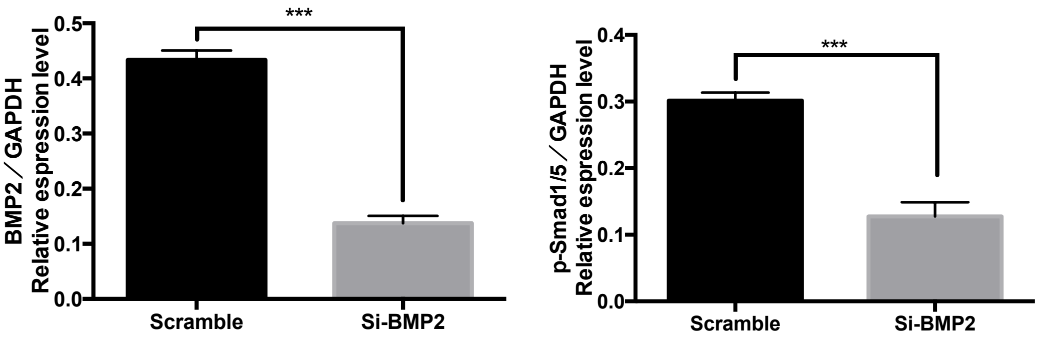

The quantification of BMP2-knockdown on the inhibition of BMP/SMAD signaling PDLSCs were transfected with 100nM BMP2 siRNA or nonrelevant siRNA (scramble). After 24h transfection, PDLSCs were incubated with osteogenic differentiation medium in the presence or absence of IL-1β (0.01ng/ml). The average ratios of BMP2/GAPDH and p-Smad1/5/GAPDH were calculated based on the analysis of the gray band intensities in western blotting at 6 days. The data are presented as the mean ± SD. **P<0.01, ***P<0.001 versus each group. All data were obtained from at least three independent experiments.

Supplementary Table 1

Primer sequence used for polymerase chain reaction amplifications.

Gene	Primer sequences	
BMP2	Forward	CCACCATGAAGAATCTTTGGA
	Reverse	GAGTTGGCTGTTGCAGGTTT
OSX	Forward	CATTCTGGGCTTGGGTATCT
	Reverse	GGCCTGAGATGAGAGTTTGT
ALP	Forward	TGGACAAGTTCCCCTTCGTG
	Reverse	CACAGATTTCCCAGCGTCCT
RUNX2	Forward	ATGCTTCATTCGCCTCACAAAC
	Reverse	CCAAAAGAAGTTTTGCTGACATGG
OPN	Forward	CTCCATTGACTCGAACGACTC
	Reverse	CAGGTCTGCGAAACTTCTTAGAT
OC	Forward	ATGAGAGCCCTCACACTCCTCG
	Reverse	GTCAGCCAACTCGTCACAGTCC
TNF-α	Forward	CCCATGTTGTAGCAAACCCTC

	Reverse	TATCTCTCAGCTCCACGCCA
IL-1ß	Forward	CCACCTCCAGGGACAGGATA
	Reverse	TGGGATCTACACTCTCCAGC
IL-6	Forward	CAATGAGGAGACTTGCCTGG
	Reverse	TGGGTCAGGGGTGGTTATTG
CCL2	Forward	CGCTCAGCCAGATGCAATCAAT
	Reverse	CTTCTTTGGGACACTTGCTGC
CCL5	Forward	CAGTCGTCCACAGGTCAAGG
	Reverse	CTTGTTCAGCCGGGAGTCAT
ß-actin	Forward	TGGCACCCAGCACAATGAA
	Reverse	CTAAGTCATAGTCCGCCTAGAAGCA
